# The effect of supplementing native rumen microbes on milk production of dairy cows

**DOI:** 10.3168/jdsc.2022-0250

**Published:** 2022-10-28

**Authors:** Katelyn Goldsmith, Josh Lefler, Mallory Embree, Michael J. VandeHaar

**Affiliations:** 1 Department of Animal Science, Michigan State University, East Lansing 48824; 2 Native Microbials Inc., San Diego, CA 92121

## Abstract

We evaluated the effects of 2 direct-fed microbial (DFM) supplements containing 4 native rumen microorganisms on the production of dairy cows. Ninety Holstein cows (43% primiparous) were fed a common diet. Mean days in milk, milk yield, and body weight at the beginning of the study (mean ± standard deviation) were 92 ± 23 d, 45 ± 10 kg/d, and 659 ± 86 kg, respectively. After 14 d, they were blocked by parity, days in milk, and energy-corrected milk (ECM) per unit of metabolic body weight. Within block, cows were randomly assigned to treatments, which were top-dressed daily for the next 112 d. Treatments were 150 g of ground corn mixed with (1) no live DFM (CON), (2) 5 g of a live DFM (Galaxis 2.0; G2), and (3) 5 g of a live DFM (Galaxis 2.0 Plus; G2P). G2 and G2P were products of Native Microbials Inc. (San Diego, CA) and contained the same organisms but in different concentrations. Supplementation with DFM did not alter yield of total milk, protein, or fat, but slightly decreased body weight gain and body condition score gain with no difference between G2 and G2P. The DFM tended to decrease dry matter intake (DMI) and tended to improve ECM/DMI. The DFM did not alter digestibility of fiber, starch, or protein and did not alter concentrations of glucose or nonesterified fatty acids, but tended to decrease concentration of insulin in plasma. Direct-fed microbials decreased somatic cell counts in milk with no difference between G2 and G2P. In conclusion, supplementation with native DFM had little impact on animal production and efficiency.

## Highlights

• Direct-fed microbial (DFM) supplementation had no effect on milk production.
• Body weight gain and BCS gain were lower in cows fed supplemental DFM.
• DFM supplementation did not alter digestibility of NDF, starch, or CP.
• DFM supplementation did not significantly alter plasma metabolite concentrations.
• DFM supplementation decreased SCC.

## Graphical Abstract

**Figure:**
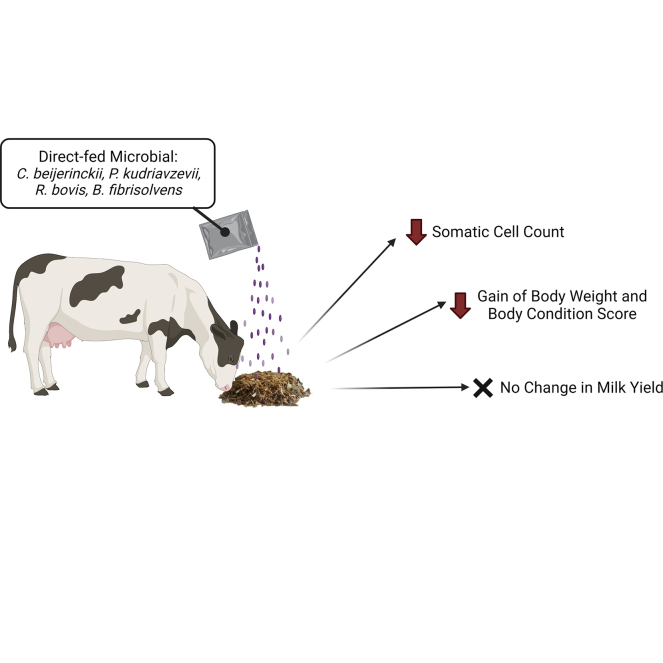
**Summary:** Direct-fed microbials (DFM) have been fed to dairy cows to improve milk production and efficiency. Direct-fed microbials commonly contain microorganisms that are not native to cows. We evaluated the effects of 2 DFM containing native rumen microorganisms on milk production of dairy cows. The supplements did not alter yield of total milk, protein, or fat, but decreased SCC and BW gain, and tended to decrease feed intake and increase energy-corrected milk/dry matter intake. Overall, native DFM treatment had little effect in this study.

Direct-fed microbials (**DFM**) are feed additives commonly used in dairy production to promote milk production, efficiency, and health. These products, composed of viable yeast and bacteria, are fed to manipulate the rumen and lower gut microbiome (Yoon and Stern, 1995). Many studies have evaluated the effect of DFM supplementation (Krehbiel et al., 2003; Chaucheyras-Durand et al., 2008). Many modes of action have been proposed for DFM, and likely include moderating rumen pH and redox potential, and improving ruminal or total-tract nutrient digestion (Yoon and Stern, 1995; Nocek et al., 2002). Direct-fed microbials sometimes increased milk production (Nocek et al., 2003; Boyd et al., 2011), but results were inconsistent and varied by DFM species, strain, dosage, frequency, and animal physiological status (Chaucheyras-Durand et al., 2008). Most organisms in commercial DFM are not native to the rumen, which may limit their ability to interact with the native microbiome (Weimer, 2015). In this study, we investigated a DFM comprising 1 yeast and 3 bacterial species isolated from the rumen of healthy dairy cattle. These species (*Clostridium beijerinckii, Pichia kudriavzevii, Ruminococcus bovis*, and *Butyrivibrio fibrisolvens*) have enhanced starch and fiber digestion in vitro (Stewart et al., 1997; Leschine, 2005; Elahi and Rehman, 2018; Gaffney et al., 2021). Our objective was to determine if supplementing dairy cows with these organisms would increase milk production and efficiency and if increased concentrations of *C. beijerinckii* and *P. kudriavzevii* would alter the response.

Ninety lactating Holstein cows at the Michigan State University Dairy Cattle Teaching and Research Center were used in 2 cohorts (cohort 1, starting November 13, 2020, n = 39, 53% primiparous; cohort 2, starting January 29, 2021, n = 51, 29% primiparous) in a randomized complete block design. Mean DIM, milk yield, and BW for all cows at the beginning of the study (mean ± SD) were 92 ± 23 d, 45 ± 10 kg/d, and 659 ± 86 kg, respectively. Within cohort, cows were blocked by parity, DIM, and ECM/BW^0.75^ and randomly assigned to treatment within block.

All experimental procedures were approved by the Michigan State University Institutional Animal Care and Use Committee.

Cows were fed a common diet for a 14-d preliminary period, and then treatments were top-dressed on the common diet for 112 d. Daily treatments were 150 g of ground corn mixed with (1) no DFM (**CON**), (2) 5 g of a live DFM (**G2**) containing *Clostridium beijerinckii* at 1.0 × 10^7^ cfu; *Pichia kudriavzevii* at 1.0 × 10^8^ cfu; *Ruminococcus bovis* at 1.0 × 10^8^ cfu; and *Butyrivibrio fibrisolvens* at 1.0 × 10^8^ cfu (Galaxis 2.0; Native Microbials Inc.), or (3) 5 g of a live DFM (**G2P**) that was similar to G2 but contained more *C. beijerinckii* (7.5 × 10^7^ cfu) and *P. kudriavzevii* (1.0 × 10^9^ cfu; Galaxis 2.0 Plus). Treatments were mixed into the top 15 cm of each cow's feed before she had access to it. Treatments were obtained from the manufacturer every 3 mo, stored at 2°C, and mixed with corn daily before feeding.

Forage DM content was determined twice weekly, and diets were adjusted accordingly. Cohort 1 was milked 3 times daily at 0730 h, 1530 h, and 2330 h before 61 d of treatment and 0530 h, 1330 h, and 2130 h thereafter. Cohort 2 was milked at 0400 h, 1200 h, and 2000 h. All cows were housed in tiestalls throughout the experiment and had access only to their own feed. Stalls were bedded with sawdust and cleaned 3 times daily. Feed was offered at 115% of expected intake once daily at 1030 h (cohort 1) or 800 h (cohort 2), and orts were recorded before feeding to adjust the amount of feed offered. Water was available ad libitum in each stall. Body weight was measured for each cow 3 d per week. Body condition was scored by 3 trained investigators on a 5-point scale in 0.25 increments (Wildman et al., 1982) at −14, 0, 28, 56, 84, and 112 d of treatment.

Daily milk yield was automatically recorded at each milking. Milk samples were collected for 6 consecutive milkings per week for component analysis. Samples were stored with preservative (Bronolab W-II liquid, Advanced Instruments) at 4°C until analysis. Individual milk samples were analyzed by CentralStar Cooperative Inc. for fat, true protein, lactose, MUN, and SCC concentrations by mid-infrared spectroscopy (AOAC, 1990, method 972.160). Milk yield and component concentrations for each milking were summed for a daily total and to calculate ECM and milk component yields. Energy-corrected milk was calculated as ECM = [(0.324 × kg of milk) + (12.816 × kg of milk fat) + (7.129 × kg of milk protein)].

Samples of all diet ingredients and TMR (~0.5 kg) were collected once weekly and stored at −20°C until composited by month and dried; these values were used for calculating final diet composition (Table 1). Apparent total-tract digestibility was determined on 10 blocks (30 cows) in each cohort over 5 d (d 29 to 34 for cohort 1 and d 35 to 40 for cohort 2). Within cohort, samples of all diet ingredients (~0.5 kg) and orts from each cow (~1.0 kg) were collected daily and composited by cow. Feces (~400 g) were sampled every 15 h over the 5 d, resulting in 8 samples/cow representing every 3 h over a day. Feces were stored at −20°C until dried and composited on an equal DM basis for each cow. Diet ingredients, orts, and fecal samples were dried at 55°C for 72 h in a forced-air oven to determine DM. Dried samples were ground with a Wiley mill (5-mm screen; Arthur H. Thomas). Samples of diet ingredients, orts, and feces were analyzed by Cumberland Valley Analytical Services (Waynesboro, PA) for CP (method 990.03; AOAC International, 2000) and starch (Hall, 2009). Ash was determined according to method 942.05 (AOAC International, 2000) modified to ash a 1.5-g sample for 4 h. The NDF and indigestible NDF were determined according to Van Soest et al. (1991) modified to use Whatman 934-AH glass micro-fiber filters with 1.5-μm particle retention (Cytiva). Indigestible NDF, estimated as NDF residue after 240 h in vitro fermentation, was used as an internal marker to predict fecal output (Cochran et al., 1986). Apparent total-tract digestibility of nutrients was calculated as in Daniel et al. (2020).

**Table 1:** Ingredient and nutrient composition of common base diet

Ingredient	% of DM	
	Cohort 1	Cohort 2
Corn silage	29.1	29.3
Alfalfa silage	13.8	14.0
Ground corn	22.4	22.6
Cottonseed, whole	7.2	7.4
Soybean meal	8.5	8.6
Soybean hulls	10.2	10.5
Vitamin-mineral mix^1^	2.1	2.2
High cow supplement mix^2^	7.6	7.7
Nutrient composition		
DM^3^	52.7	54.3
NDF	29.2	28.9
Forage NDF	18.2	20.5
Starch	26.9	27.7
CP	16.8	16.8
RUP^4^	33.7	34.0

1 Vitamin and mineral mix contained 22.0% fine ground corn grain, 20.0% calcium carbonate, 19.1% calcium phosphate, 10.0% sodium chloride, 4.6% sodium sesquinate, and trace minerals and vitamins to meet NRC (2001) requirements.

2 High cow supplement mix contained 39.5% Amino Plus (Ag Processing Inc.), 18.4% Caledonia Pass (Caledonia Farmers Elevator), 15.8% sodium sesquicarbonate, 12.8% calcium carbonate, 8.7% fine ground corn grain, 2.7% urea, and 1.1% Smartamine M (Adisseo).

3 Expressed as percent of as fed.

4 Expressed as percent of CP based on NASEM (2021).

From each cow in the digestibility study, blood (~15 mL) was sampled at each fecal collection and on 93 ± 1 d at −1, +2, and +6 h after feeding into EDTA- and sodium fluoride-coated tubes. Plasma was harvested after centrifugation at 2,000 × *g* for 15 min at 4°C and stored at −20°C until composited by cow and analyzed. Plasma nonesterified fatty acid (**NEFA**), glucose, and insulin concentrations were analyzed using commercially available kits according to the manufacturer's instructions (NEFA: Sekishui Diagnostics; glucose: Sigma-Aldrich; insulin: Mercodia). Absorbance was measured with a micro-plate reader (SpectraMax 190; Molecular Devices Corp.).

All data were analyzed using the mixed model of SAS (version 9.4; SAS Institute Inc.) according to the following model:

Y_ijkl_ = Cov + μ + C_i_ + P_j_ + C_i_′P_j_ + B_k_(C_i_′P_j_) + T_l_ + T_l_′C_i_ + T_l_′P_j_ + T_l_′C_i_′P_j_ + e_ijkl_,

where Y_ijkl_ = dependent variable, Cov = fixed effect of 2-week covariate period, μ = overall mean, C_i_ = fixed effect of cohort (i = 1 to 2), P_j_ = fixed effect of parity (j = 1 to 2), C_i_′P_j_ = interaction of cohort and parity, B_k_(C_i_′P_j_) = random effect of block within cohort and parity, T_l_ = fixed effect of treatment (l = 1 to 3), T_l_′C_i_ = interaction of treatment and cohort, T_l_′P_j_ = interaction of treatment and parity, T_l_′C_i_′P_j_ = interaction of treatment, cohort, and parity, and e_ijkl_ = residual error. Week on treatment was originally included in the model but deleted because it was not significant. Body weight gain/d and BCS gain/28 d were analyzed using the same model but excluding the covariate variable. Normality of results was tested using box plots, normal probability, and homogeneity of variances. Somatic cell count displayed nonnormality so was log-transformed for analysis. Main effects were declared significant at *P* ≤ 0.05 and tendencies at 0.05 < *P* ≤ 0.15. All data were expressed as LSM and SEM, unless otherwise specified. Preplanned contrasts were CON versus DFM and G2 versus G2P.

On average, cows produced 44 kg of milk daily with 3.9% fat, 3.2% true protein, 4.9% lactose, and 13 mg/mL of MUN (Table 2). Yields of milk, protein, fat, lactose, MUN, and ECM were not affected by treatment (all *P* > 0.4). Treatment tended to decrease milk fat concentration slightly in cohort 2 but not in cohort 1 (*P* = 0.07 for T′C interaction). Average SCC of CON was 20,700 cells/mL; DFM decreased SCC (*P* = 0.05), but G2 and G2P were not different (*P* = 0.28). Average BW was 690 kg and BCS was 3.2. Treatments altered BW gain (0.51 kg/d for CON vs. 0.38 kg/d for DFM; *P* < 0.001) and BCS gain (0.70 units/28 d for CON vs. 0.05 units/28 d for DFM; *P* = 0.04). The effect for BW gain was most pronounced in cohort 1 (*P* = 0.02 for T′C interaction) where animals fed the DFM treatment on average gained 0.19 kg/d less than CON in cohort 1 but 0.07 kg/d less than CON in cohort 2. No differences between G2 and G2P were detected for gain in BW or BCS (both *P* > 0.7).

**Table 2:** Effects of supplementation with native rumen microbes on milk production, BW, and blood measures^1^

Variable	Treatment			SEM	*P*-value	
	CON	G2	G2P		CON vs. DFM	G2 vs. G2P
DMI (kg)	29.6	29.3	28.8	0.28	0.08	0.14
Milk (kg/d)	43.8	44.4	44.2	0.52	0.46	0.75
ECM^2^ (kg/d)	46.1	45.9	45.7	0.51	0.60	0.73
Fat (%)	3.90	3.85	3.87	0.053	0.37	0.77
Fat (kg/d)	1.69	1.68	1.68	0.023	0.47	0.89
Protein (%)	3.21	3.19	3.19	0.017	0.23	0.88
Protein (kg/d)	1.41	1.40	1.39	0.018	0.58	0.52
Lactose (%)	4.92	4.93	4.92	0.007	0.37	0.47
Lactose (kg/d)	2.27	2.17	2.20	0.954	0.94	0.52
MUN (mg/dL)	13.0	13.0	12.9	0.02	0.89	0.57
SCC^3^ (×1,000 cells/mL)	20.7	13.5	16.5	2.18	0.05	0.28
Feed efficiency (ECM/DMI)	1.54	1.57	1.58	0.016	0.06	0.43
Captured energy/feed energy	0.27	0.27	0.27	0.003	0.52	0.33
BW (kg)	694	687	686	2.5	0.02	0.61
BW gain (kg/d)	0.508	0.375	0.380	0.0289	0.001	0.89
BCS	3.20	3.14	3.16	0.021	0.05	0.48
BCS gain (unit/28 d)	0.070	0.052	0.048	0.0077	0.04	0.74
Glucose (mg/dL)	55.5	54.6	54.5	0.79	0.34	0.91
NEFA^4^ (μ*M*)	90.4	81.2	92.4	3.49	0.41	0.031
Insulin (μg/L)	0.947	0.812	0.851	0.0573	0.057	0.57

1 Treatments were (1) control (CON); (2) 5 g of Galaxis 2.0 (G2; Native Microbials Inc.); (3) 5 g of Galaxis 2.0 Plus (G2P; Native Microbials Inc.). Blood samples composited from collections every 15 h over 5 d.

2 ECM = [(0.324 × kg of milk) + (12.95 × kg of milk fat) + (7.20 × kg of milk protein)].

3 Due to nonnormality, SCC was log-transformed for analysis. Means were back transformed for reporting. SEM were calculated as the difference between back-transformed means and back-transformed means ± SEM.

4 NEFA = nonesterified fatty acids.

Average DMI of CON cows was 29.6 kg/d, and DFM tended to decrease DMI by 0.6 kg/d (*P* = 0.08) with the decrease tending to be greater in G2P than G2 (*P* = 0.14). The DFM tended to increase ECM/DMI (*P* = 0.06) from 1.54 to 1.58, and this difference was due to cohort 1; ECM/DMI was 1.55 for CON and 1.61 for DFM in cohort 1 but 1.54 for both CON and DFM in cohort 2 (*P* = 0.14 for T′C interaction). No difference was detected between G2 and G2P. The DFM did not improve captured energy over feed energy (*P* > 0.5). Apparent total-tract digestibility of OM tended (*P* = 0.06, Table 3) to decrease with DFM, and G2 tended to have a greater effect than G2P (*P* = 0.15). Apparent total-tract digestibilities of starch, NDF, and CP were not altered by treatment (all *P* > 0.15).

**Table 3:** Effect of supplementation with native direct-fed microbial on apparent total-tract digestibility of nutrients

Variable	Treatment^1^			SEM	*P*-value	
	CON	G2	G2P		CON vs. DFM	G2 vs. G2P
DMI (kg)	29.6	29.3	28.8	0.28	0.08	0.14
OM digestibility (%)	66.1	64.8	65.6	0.44	0.06	0.15
NDF digestibility (%)	45.4	43.7	45.1	0.75	0.19	0.12
Starch digestibility (%)	98.5	98.5	98.4	0.18	0.88	0.66
CP digestibility (%)	60.8	59.8	60.6	0.75	0.49	0.48

1 Treatments were (1) control (CON); (2) 5 g of Galaxis 2.0 (G2; Native Microbials Inc.); (3) 5 g of Galaxis 2.0 Plus (G2P; Native Microbials Inc.).

Average plasma glucose, NEFA, and insulin concentrations were 55 mg/dL, 88 μ*M*, and 0.87 μg/L, respectively (Table 2). Treatment with DFM tended to decrease plasma insulin concentration by 11% (*P* = 0.06), but the interaction of cohort and treatment also tended to be different (*P* = 0.07) with a 24% decrease in cohort 1 but a 3% increase in cohort 2. No difference in plasma insulin was observed for G2 compared with G2P (*P* = 0.6). Compared with CON, DFM treatments did not alter plasma NEFA concentration (*P* = 0.4) but G2P had greater NEFA concentration than G2 (*P* = 0.03). Plasma glucose concentration was not affected by treatment (*P* = 0.3). There was no significant interaction of cohort and treatment for plasma glucose and NEFA concentrations (both *P* > 0.2). All 3 blood measures were affected by time relative to feeding (*P* < 0.01, data not shown). For times −1, +2, and +6 h relative to feeding, plasma insulin averaged 0.44, 1.10, and 0.87 μg/L, plasma glucose was 58, 51, and 54 mg/dL, and plasma NEFA was 84, 70, and 73 μ*M*, respectively. The DFM treatments tended (*P* = 0.12) to decrease NEFA after feeding relative to controls but had no effect on feeding responses for insulin and glucose.

Based on the tendency for lower OM digestibility and DMI of cows fed the native DFM, we suggest the native DFM altered rumen fermentation to decrease the postfeeding spikes in propionate and caused the trend for less plasma insulin. Lower concentrations of plasma insulin may have resulted in partitioning available energy toward milk production instead of body tissue and thus reducing gains with no drop in milk yield. Additionally, DFM did not alter plasma NEFA concentrations indicating fat mobilization was not altered to sustain production. The lack of effect on milk production and composition in this study is similar to a study by Goetz et al. (2021) who fed 2 of the same species as this study, *C. beijerinckii* and *P. kudriavzevii*, and also saw no change in production.

The organisms *R. bovis* and *C. beijerinckii* were expected to enhance starch digestion (Leschine, 2005; Gaffney et al., 2021). Perhaps the reason we saw no improvement in digestion or production from native DFM in our study was because our diet was high in starch (27%) from highly digestible sources with 98.5% total-tract digestibility in the control diet. Ruminal starch digestibility was likely lower than 98.5% but perhaps further improvements in starch digestion were unlikely.

In conclusion, supplementation of these DFM supplements significantly decreased gains in BW and BCS, decreased SCC, and tended to decrease DMI, SCC, OM digestion, plasma insulin concentration, and increase ECM/DMI. Effects tended to be greater in cohort 1 than cohort 2. Milk production and composition, as well as digestion of several nutrients (NDF, starch, and CP) and concentrations of plasma metabolites (glucose, NEFA) were unaffected.
